# Ewing sarcoma of the frontal sinus in an elderly patient: a case report and review

**DOI:** 10.1007/s13691-026-00849-3

**Published:** 2026-03-21

**Authors:** Risako Hagiwara, Yosuke Ariizumi, Yumiko Tateishi, Susumu Kirimura, Takahiro Asakage

**Affiliations:** 1 Department of Otorhinolaryngology, Institute of Science Tokyo, Tokyo, Japan; 2 Department of Head and Neck Surgery, Institute of Science Tokyo, 1-5-45 Yushima, Bunkyo-ku, Tokyo, 113-8510 Japan; 3 Division of Pathology, Institute of Science Tokyo Hospital, Tokyo, Japan

**Keywords:** Ewing sarcoma, Olfactory neuroblastoma, Frontal sinus tumor, Elderly patient, Sinonasal small round cell tumor, Carbon-ion radiotherapy

## Abstract

Ewing sarcoma is a malignant small round cell tumor with an aggressive clinical course that typically arises in the extremities or trunk of adolescents and young adults. Sinonasal Ewing sarcoma in elderly patients is extremely rare, and its diagnosis can be challenging owing to overlapping histopathological features with other malignancies. A 71-year-old woman with a sinonasal tumor was referred to our hospital. The initial pathological diagnosis based on an outpatient biopsy was olfactory neuroblastoma (ONB). However, the absence of tumor in the olfactory cleft suggested other histological types. A surgical biopsy was performed, and Ewing sarcoma was confirmed by immunostaining and fluorescence in situ hybridization. One month after surgical biopsy, rapid tumor growth with intracranial extension was observed. Chemotherapy and extensive skull-base surgery were declined. Although carbon-ion radiotherapy was effective for the primary tumor, bone metastases developed shortly after treatment, and the patient died 7 months after the initial visit. This case highlights the diagnostic pitfalls of sinonasal malignancies and the importance of endoscopic and radiologic findings as potential diagnostic clues. In frontal sinus Ewing sarcoma, local treatment alone is unlikely to achieve cure without chemotherapy. However, the standard regimen for Ewing sarcoma is often too toxic for elderly patients, underscoring the need for more tolerable treatment options. To our knowledge, this is the first reported case of frontal sinus Ewing sarcoma in a patient over 60 years old.

## Introduction

Ewing sarcoma is a rare sarcoma of bone or soft tissue, occurring at an incidence of approximately 3 cases per million annually [1]. First described in 1920 by James Ewing, it was recognized as a distinct entity from osteosarcoma due to its unique morphology and radiosensitivity. Advances in molecular and immunohistochemical (IHC) testing later confirmed that osseous and extraosseous Ewing sarcoma, peripheral primitive neuroectodermal tumor (pPNET), and Askin’s tumor share common genetic features, particularly Ewing sarcoma breakpoint region 1 (*EWSR1*) rearrangements, and are now collectively referred to as the Ewing sarcoma family of tumors [2].

Ewing sarcoma typically arises in the extremities and trunk of adolescents and young adults [3, 4]. Only a limited number of sinonasal cases have been reported, and their clinical presentation and diagnostic features remain poorly characterized. In the sinonasal region, because a wide spectrum of small round cell tumors arise, achieving an accurate histopathological diagnosis can be challenging in certain cases. Although Ewing sarcoma is highly aggressive, extended surgery for skull base invasion or intensive chemotherapy is often unfeasible in elderly patients, and the optimal treatment strategies for this population have not yet been established.

We report a case of Ewing sarcoma in the frontal sinus of an elderly patient, initially diagnosed as olfactory neuroblastoma (ONB). To the best of our knowledge, this is the first reported case of Ewing sarcoma arising from the frontal sinus in patients over 60 years old.

## Case report

**Fig. 1:**
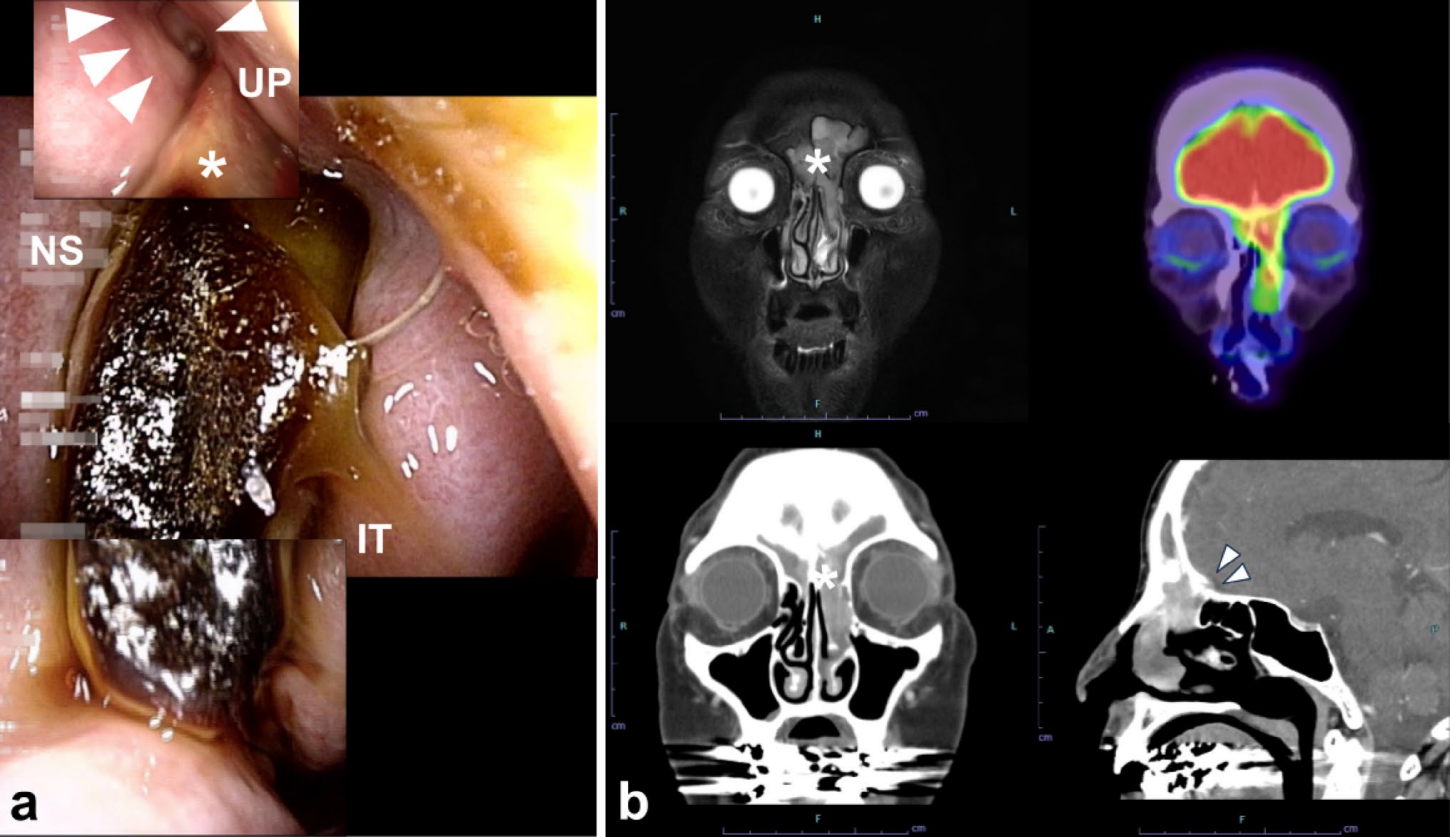
Nasal endoscopy (**a**), MRI, CT, and PET/CT (**b**) at initial presentation

**Fig. 2:**
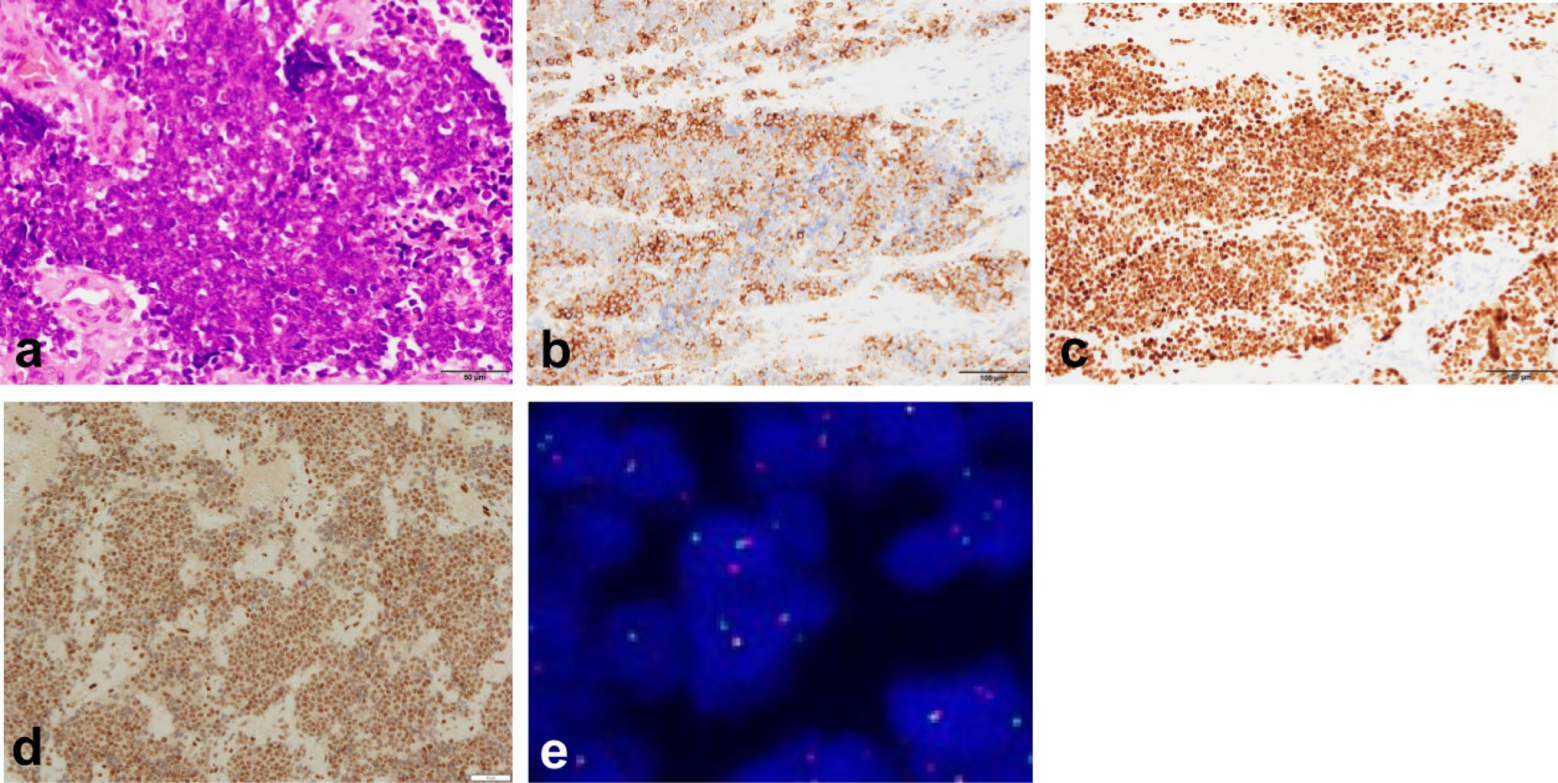
Pathological findings (**a**) H&E staining: The tumor is comprised of small round cells with a high N/C ratio, proliferating in solid nests surrounded by thick vascular fibrous stroma. scattered mitotic figures and apoptotic bodies can be seen. (**b**) Synaptophysin immunostaining: Synaptophysin positivity mimicking olfactory neuroblastoma. (**c**) NKX2.2 immunostaining: NKX2.2 positivity suggesting Ewing sarcoma.(**d**) FLI-1 immunostaining: FLI-1 positivity is consistent with Ewing sarcoma. (**e**) EWSR1 FISH: EWSR1 split signal was detected in 72% of cells (control: 1.7%), confirming EWSR1 rearrangement

**Fig. 3:**
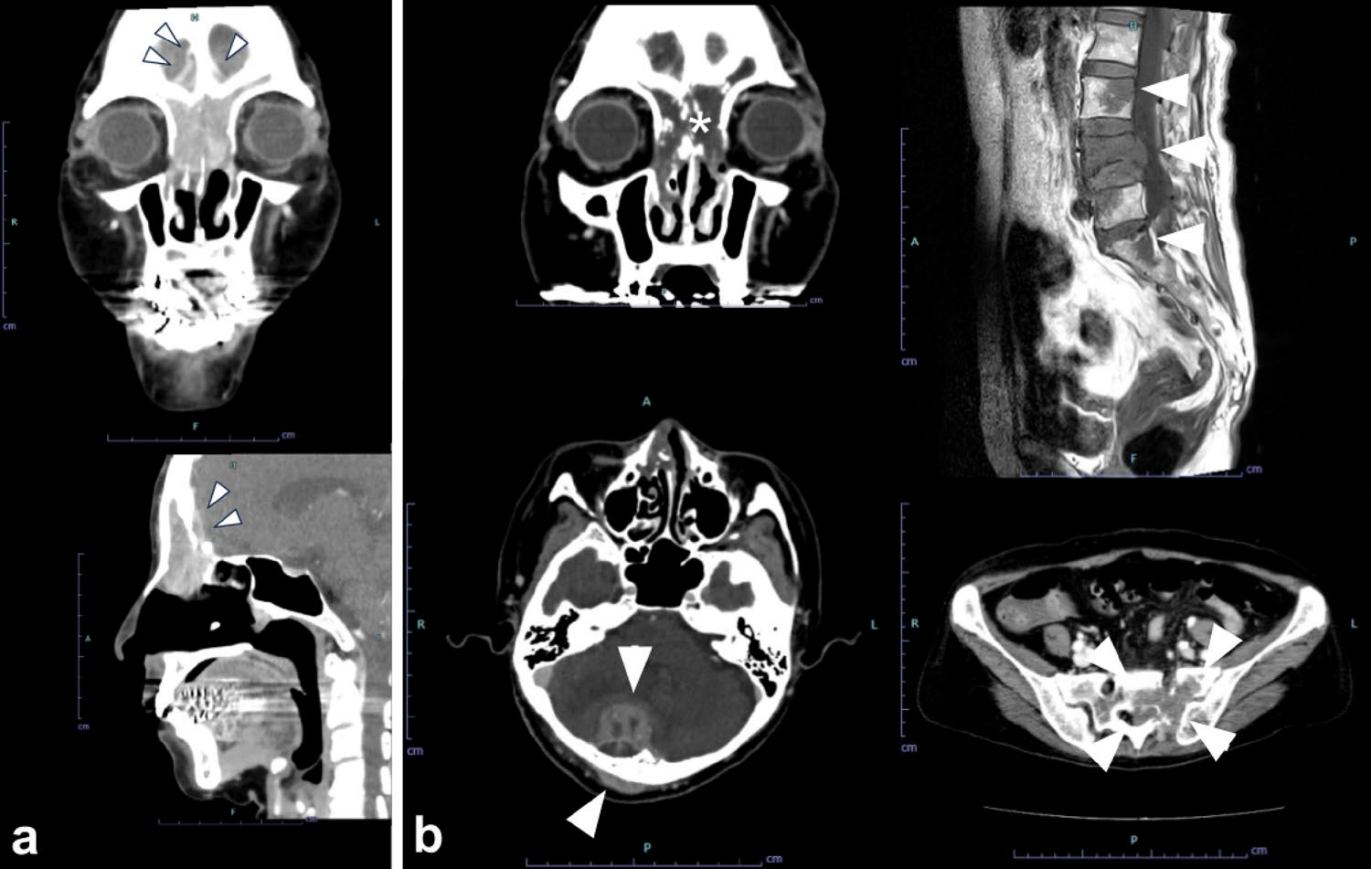
Imaging findings of the postoperative course (**a**) One month after surgery, the tumor rapidly regrew with evident intracranial invasion (arrowheads). (**b**) Following carbon-ion radiotherapy, contrast enhancement of the tumor has diminished (asterisk in coronal images); however, at five months postoperatively, multiple bone metastases, including the occipital bone, pelvic bones, and spine, are observed

A 71-year-old woman with a history of breast cancer treated with surgery and chemoradiotherapy 10 years previously presented with purulent nasal discharge and epistaxis. She visited a nearby hospital. A tumor filling the left nasal cavity was identified. The initial biopsy from the anterior surface of the tumor suggested olfactory neuroblastoma (ONB), with synaptophysin positivity indicating neuroendocrine differentiation and scattered S-100 positive cells in the background. She was referred to our department two months after the onset of symptoms. Nasal endoscopy revealed a tumor protruding into the nasal cavity from the middle meatus (Fig. 1). Contrast-enhanced CT and MRI showed a 28 × 17 × 38 mm tumor centered in the left ethmoid and frontal sinuses, without invasion of the olfactory cleft. Bone erosion of the posterior wall of the frontal sinus was noted, but no clear intracranial invasion was observed. PET/CT showed ^18^FDG accumulation (SUVmax 8.5) at the primary site only, with no lymph node or distant metastasis (Fig. 1). Although the previous pathological diagnosis was ONB, the absence of tumor in the olfactory cleft suggested an alternative histological diagnosis. Consultation with a pathologist determined that it would be necessary to assess the entire tumor for an accurate diagnosis, and a surgical biopsy under general anesthesia was planned. To expose the tumor without damaging it, the bases of the frontal sinuses were opened with the modified endoscopic Lothrop procedure (outside-in approach). The left frontal and ethmoid sinus were occupied by a hemorrhagic tumor. The nasal and ethmoid mucosa and bones were not involved. Infiltration of mucosa or bone was found only in a small focus in the center of the posterior wall of the frontal sinus, thought to be the origin of the tumor. When traction was applied on the tumor margin in order to obtain adequate specimens for pathological examination, most of the tumor detached, leaving a 5-mm residual focus. The pathological examination with hematoxylin & eosin staining showed a small round-cell tumor, with a high nucleo-cytoplasmic ratio, showing round cells forming a vesicular-like cluster surrounded by a thick vascular fibrous stroma. These histological features are characteristic of small round cell tumors. Comprehensive immunohistochemistry (IHC) revealed positive NKX2.2 immunostaining, a lack of S100-positive sustentacular cells, and FLI-1 positivity, suggesting Ewing sarcoma. The diagnosis was confirmed by *EWSR1* fluorescence in situ hybridization (FISH) (Fig. 2). Due to the exhaustion from previous chemotherapy for breast cancer and her advanced age, she declined chemotherapy and extended resection; therefore, postoperative radiotherapy was planned. Five weeks after surgical biopsy, the residual tumor regrew from 5 mm to 42 mm with intracranial extension (Fig. 3a). The planned postoperative radiotherapy was canceled, and definitive carbon-ion radiotherapy at a dose of 70.4 Gy was administered 2 to 3 months after the surgical biopsy. MRI showed partial reduction of the tumor. Genetic panel testing detected the Ewing sarcoma breakpoint region 1 *(EWSR1-FLI-1)* fusion gene; however, no actionable therapeutic targets were identified. Two months later, at five months postoperatively, the primary site had shrunk and remained stable, but multiple bone metastases appeared (Fig. 3b). Due to spinal metastasis, she developed sensory and motor impairment in both lower limbs, and her ECOG performance status worsened to 3. She died seven months after her initial visit to our hospital and two months after the onset of distant metastasis.

The left nasal cavity is involved by tumor (asterisk) protruding from the middle meatus, while the olfactory cleft remains intact (white arrows). The tumor surface is covered with crusts from the previous biopsy. The nasal septum (NS), uncinate process (UP), and inferior turbinate (IT) are not infiltrated. The middle turbinate is obscured by the tumor. MRI and CT show a 28 × 17 × 38 mm tumor (asterisk) extending across the left ethmoid and bilateral frontal sinuses, without involvement of the olfactory cleft. The frontal sinus is filled with tumor and retained fluid. Slight intracranial invasion via the posterior wall of the frontal sinus was suspected (arrowheads). No metastases were observed on PET/CT.

## Discussion

A case of Ewing sarcoma in the frontal sinus of an elderly patient is presented. This case highlights two key considerations: first, sinonasal Ewing sarcoma may be misdiagnosed as other small round cell tumors, such as ONB; second, surgery or carbon-ion radiotherapy alone is unlikely to achieve curative outcomes without chemotherapy.

Making a pathological diagnosis of sinonasal tumor is not easy. Sinonasal tumors are often hemorrhagic and present with surrounding inflammatory mucosa. The limited amount of tumor tissue obtainable in an outpatient setting may not allow for the comprehensive staining needed to differentiate various tumors. In this case, the initial pathological diagnosis was ONB; however, endoscopy and imaging showed no tumor in the olfactory cleft, raising clinical suspicion of an alternative histological type. A larger tissue sample was eventually obtained under general anesthesia, enabling detailed histological evaluation with extensive IHC, and a definitive diagnosis of Ewing sarcoma was established by FISH. Ewing sarcoma is most common in adolescence or early adulthood, with only 6%–8.5% [4, 5] of cases in patients > 40 y and 1% in those > 60 y [4]. However, among 93 reported cases of sinonasal Ewing sarcoma [6–12], 7 (7%) were patients over 60 years old, suggesting a higher proportion of elderly patients in sinonasal cases compared with other sites(Table 1). Sinonasal Ewing sarcoma presents as a rapidly progressing tumor with nonspecific symptoms, such as nasal obstruction, epistaxis, and visual impairment. It has no characteristic imaging findings [6]. Ewing sarcoma is classified as a small blue round-cell tumor that may arise in the sinonasal cavities, along with other tumors such as ONB, neuroendocrine carcinoma, sinonasal undifferentiated carcinoma, rhabdomyosarcoma, malignant melanoma, and lymphoma [13]. The current definition of Ewing sarcoma encompasses sarcomas of small round cells that possess fusion between a member of the *FET* (typically *EWSR1*) and *ETS* gene families [2]. ONB is a sinonasal malignancy arising from the olfactory epithelium in the olfactory cleft. The predominant age range for ONB is between 40 and 60 y [14]. Misdiagnosis of ONB as other small round-cell tumors has been reported [15]. Currently, IHC and FISH assays facilitate the differential diagnosis. FLI-1 is positive in about 70% of Ewing sarcomas, whereas it is negative in nearly all ONBs [16]. Detection of a fusion gene is definitive for diagnosing Ewing sarcoma [2]. When Ewing sarcoma is not suspected, fusion gene testing may not be performed, leading to a misdiagnosis as ONB or other histological types. In cases where the pathological diagnosis conflicts with clinical findings, alternative histological types should be considered, and further pathological examination should be performed.

**Table 1:** Reported cases of sinonasal ewing sarcoma in patients aged over 60 years

Author	Age	Gender	Primary site	Tumor extension	Initial Treatment	Observation period (Months)	Outcome
Shah 2014	67	Male	Maxillary sinus	None	Partial maxillectomy Vincristine and cisplatin	17	Lymph node and liver metastases Dead of disease
Dutta 2014	67	Male	Maxillary sinus	Cheek subcutaneous tissue	Total maxillectomy VDC-IE RT	2	No recurrence
Lee 2018	68	Female	Ethmoid sinus	Extraocular muscles and optic nerve	VDC-IE RT Docetaxel and gemcitabine	12	No recurrence
Hafezi 2011	69	Female	Maxillary sinus and nasal cavity	Oral cavity	Chemoradiation	6	No recurrence
Hafezi 2011	70	Female	Nasal cavity	Infratemporal fossa	Surgery and postoperative RT	21	No recurrence
Worthy 2023	89	Male	Ethmoid sinus	Brain	Palliative care	-	Dead of disease
Kulkarni 2016	70	Female	Maxillary sinus	Orbital floor	Not available	-	Lost to follow-up
Hagiwara 2026 Present case	71	Female	Frontal sinus	Dura	Carbon-ion radiotherapy	7	Bone metastasis Dead of disease

VDC-IE: chemotherapy with vincristine, doxorubicin, and cyclophosphamide, alternating with cycles of ifosfamide and etoposide; RT: radiotherapy

No case had lymph nodes or distant metastases at initial presentation.

Ewing sarcoma is highly metastatic. The standard treatment is a multimodality approach that combines systemic chemotherapy with local therapies such as surgery and radiotherapy. The chemotherapy regimen consists of vincristine, doxorubicin, and cyclophosphamide, alternating with cycles of ifosfamide and etoposide (VDC-IE). This regimen is highly toxic, making it difficult for many elderly patients to maintain dose intensity, leading to poorer outcomes [17]. Older patients are more likely to require dose reductions, particularly for doxorubicin, due to myelosuppression [18]. A subgroup analysis of a randomized controlled trial found the benefit of adding IE only in patients aged ≤ 17 y [19].

A case of Ewing sarcoma in the frontal sinus of an elderly patient was presented. The tumor initially was suspected to be ONB. While clinical information—such as the patient’s age, endoscopic appearance, and radiological findings—is generally important for pathological diagnosis, it is particularly essential for evaluating sinonasal small round-cell tumors. For sinonasal Ewing sarcoma, surgery followed by radiotherapy or definitive carbon-ion radiotherapy are insufficient. In elderly patients, intensive chemotherapy can be challenging. Developing optimal treatment strategies for elderly patients is therefore highly desirable.

## Data Availability

The data supporting the findings of this case report are available from the corresponding author upon reasonable request.
